# Plasminogen activator inhibitor-1 promotes immune evasion in tumors by facilitating the expression of programmed cell death-ligand 1

**DOI:** 10.3389/fimmu.2024.1365894

**Published:** 2024-05-08

**Authors:** Abd Aziz Ibrahim, Taku Fujimura, Tomoko Uno, Tomoya Terada, Ken-ichi Hirano, Hiroyuki Hosokawa, Akio Ohta, Toshio Miyata, Kiyoshi Ando, Takashi Yahata

**Affiliations:** ^1^ Translational Molecular Therapeutics Laboratory, Division of Host Defense Mechanism, Tokai University School of Medicine, Kanagawa, Japan; ^2^ Department of Dermatology, Tohoku University Graduate School of Medicine, Sendai, Japan; ^3^ Department of Hematology and Oncology, Tokai University School of Medicine, Kanagawa, Japan; ^4^ Department of Immunology, Tokai University School of Medicine, Kanagawa, Japan; ^5^ Department of Immunology, Institute of Biomedical Research and Innovation, Foundation for Biomedical Research and Innovation at Kobe, Kobe, Japan; ^6^ Department of Molecular Medicine and Therapy, United Centers for Advanced Research and Translational Medicine, Tohoku University Graduate School of Medicine, Miyagi, Japan

**Keywords:** PAI-1, PD-L1, tumor immunity, tumor immune evasion, immune check point, small compound inhibitor

## Abstract

**Background:**

Increased levels of plasminogen activator inhibitor-1 (PAI-1) in tumors have been found to correlate with poor clinical outcomes in patients with cancer. Although abundant data support the involvement of PAI-1 in cancer progression, whether PAI-1 contributes to tumor immune surveillance remains unclear. The purposes of this study are to determine whether PAI-1 regulates the expression of immune checkpoint molecules to suppresses the immune response to cancer and demonstrate the potential of PAI-1 inhibition for cancer therapy.

**Methods:**

The effects of PAI-1 on the expression of the immune checkpoint molecule programmed cell death ligand 1 (PD-L1) were investigated in several human and murine tumor cell lines. In addition, we generated tumor-bearing mice and evaluated the effects of a PAI-1 inhibitor on tumor progression or on the tumor infiltration of cells involved in tumor immunity either alone or in combination with immune checkpoint inhibitors.

**Results:**

PAI-1 induces PD-L1 expression through the JAK/STAT signaling pathway in several types of tumor cells and surrounding cells. Blockade of PAI-1 impedes PD-L1 induction in tumor cells, significantly reducing the abundance of immunosuppressive cells at the tumor site and increasing cytotoxic T-cell infiltration, ultimately leading to tumor regression. The anti-tumor effect elicited by the PAI-1 inhibitor is abolished in immunodeficient mice, suggesting that PAI-1 blockade induces tumor regression by stimulating the immune system. Moreover, combining a PAI-1 inhibitor with an immune checkpoint inhibitor significantly increases tumor regression.

**Conclusions:**

PAI-1 protects tumors from immune surveillance by increasing PD-L1 expression; hence, therapeutic PAI-1 blockade may prove valuable in treating malignant tumors.

## 1 Introduction

The correlation between the fibrinolytic system and cancer progression has been widely acknowledged (1–3). The central factors in this mechanism include the urokinase plasminogen activator (uPA), the uPA receptor (uPAR), and the uPA inhibitor, plasminogen activator inhibitor-1 (PAI-1). Given the evidence linking increased tumor uPA expression with reduced overall survival rates and the consequent inhibitory effect of PAI-1 on uPA, PAI-1 was hypothesized to possess anti-tumor properties that delay cancer progression (4, 5). However, paradoxically, high levels of PAI-1 have been found to correlate with poor prognosis in various cancers; this is known as the “PAI-1 paradox” (6–8). Overexpression of PAI-1 in various tumors is a strong predictor of poor clinical outcomes and poor response to therapy (9, 10). Indeed, PAI-1 is a multifunctional protein that regulates fibrinolysis as well as cell proliferation, migration, and apoptosis (11–13). Furthermore, after being produced by various cell types within the tumor microenvironment, including tumor cells, adipocytes, macrophages, fibroblasts, smooth muscle cells, and endothelial cells (10), PAI-1 plays autocrine and paracrine roles in tumorigenesis (14). While ample data suggest a link between PAI-1 and cancer, its precise impact on cancer progression remains under debate.

Programmed cell death ligand-1 (PD-L1) binds to its receptor, programmed cell death protein-1 (PD-1), and inhibits T lymphocyte proliferation, cytokine production, and cytolytic activity, suppressing the immune response (15, 16). Although this mechanism helps to counteract autoimmune disease pathogenesis, it also hinders the ability of immune cells to eliminate tumor cells (17, 18). Unlike PD-1, which is primarily expressed on immune cells, PD-L1 is expressed on tumor cells and surrounding cells, including tumor-associated macrophages (TAMs) and cancer-associated fibroblasts (CAFs) (19, 20). Hence, PD-L1 plays a significant role in evading tumor immune responses, with several transcription factors that regulate its transcriptional activation (21). The JAK/STAT pathway involves a key transcription factor that binds to the PD-L1 promoter and regulates PD-L1 expression (18, 22).

Although abundant data support the involvement of PAI-1 in cancer progression, whether PAI-1 contributes to tumor immune surveillance remains unclear. According to a recent study, PAI-1 induces the internalization of PD-L1 for surface lysosomal degradation, thus downregulating PD-L1 plasma membrane expression in melanoma cells (23). The precise influence of PAI-1 on cancer progression involves tumor-promoting and anti-tumor effects. Several studies have examined the intracellular signaling pathways related to PAI-1 activity and revealed that PAI-1 activates the JAK/STAT pathway by binding to low-density lipoprotein receptor-related protein 1 (LRP1) (24, 25). This study aims to evaluate the effect of PAI-1 on PD-L1 expression in tumor cells and elucidate the underlying mechanisms of tumorigenesis, thereby defining PAI-1 as a molecular target for cancer therapy.

## 2 Methods

### 2.1 Reagents

A tPA-dependent hydrolysis assay demonstrated that TM5614 functioned as a PAI-1 inhibitory compound and displayed significant inhibition of PAI-1 activity, with an IC50 value < 6.95 μM (26, 27). The STAT3 inhibitors BP-1-102, 4-(N-(4-cyclohexylbenzyl)-2-(2,3,4,5,6-pentafluoro-N-methylphenylsulfonamido)acetamido)-2-hydroxybenzoic acid, and C188-9; N-(1′,2-dihydroxy-1,2′-binaphthalen-4′-yl)-4-methoxybenzenesulfonamide were procured from Sigma-Aldrich (St. Louis and Burlington, MA, USA). Please refer to **Supplementary Table S1** for relevant information on the antibodies used in this study.

### 2.2 Cell lines

B16 (murine melanoma; F1 (parental), F10 (metastatic)), HEK293T (human embryonic kidney), ES2 (human clear cell ovarian carcinoma), and MOLM14 (human acute myeloid leukemia) cell lines were obtained from the American Type Culture Collection (ATCC, Manassas, VA, USA). The MC38 (murine colon adenocarcinoma) cell line was procured from Kerafast. The cells were cultured in either RPMI-1640 medium (FujiFilm Wako Pure Chemical, Osaka, Japan) for HEK293T, MOLM14, and K562 cells or DMEM (FujiFilm Wako Pure Chemical) for B16F1, B16F10, MC38, and ES2 cells supplemented with 10% heat-inactivated fetal bovine serum (FBS, Biosera, Cholet, France) and antibiotics (100 U penicillin/mL and 100 μg streptomycin/mL, Thermo Fisher Scientific, Waltham, MA, USA) at 37°C in a humidified atmosphere containing 5% CO_2_.

### 2.3 Generation of gene-modified cells

The 32D cells—IL-3-dependent murine myelomonocytic progenitor cells—were transduced using a retrovirus carrying the human BCR/ABL vector to generate chronic myeloid leukemia-like cells, i.e., 32Dp210 cells. To select IL-3-independent or BCR/ABL-dependent leukemic cells, the cells were cultured in an IL-3-free medium 48 h post-infection and subsequently cloned by limiting dilution. The murine PAI-1-*ires*-GFP vector was lentiviral-transduced into 32Dp210, HEK293T, ES2, K562, and MC38 cells; 48 h after infection, cells expressing GFP were sorted and plated using clonal limit dilution. Clones that expressed PAI-1 were verified via real-time quantitative reverse-transcription polymerase chain reaction (qRT-PCR) (hereafter designated PAI-1 OE). Pairs of chimeric sgRNA-specific oligos were selected using public online tools (crispr.mit.edu) to generate genomic deletions in uPAR and LRP1. The sgRNA-specific oligos were phosphorylated, annealed, and integrated into spCas9 plasmids (pX459; Addgene, Watertown, MA, USA). The cells were transfected with paired pX459-sgRNA plasmids via electroporation. Transfected cells with resistance to 2 μg/mL puromycin were selected and cloned through limiting dilution. Monoallelic and biallelic deletion clones were validated using conventional PCR and Sanger sequencing. The following PCR primer sequences were used: PAI-1, 5′-GGGTTCACTTTACCCCTCCG-3′ and 5′-TATCGCAGCACCAGAGTCAC-3′; LRP1, 5′-CTGAGAAAGCTGCCTACTGT-3′ and 5′-ATCGGTCCAGTACACATTCC-3′; uPAR, 5′-AACTCAGCCTCATTGCCTCT-3′ and 5′-AATTCCCTGCTGCTCTCATC-3′. Downregulation of target gene expression was confirmed via qRT-PCR analysis using the appropriate TaqMan primers/probes. The levels of PAI-1, Mm00435860_m1, PD-L1, Mm00452054_m1, LRP-1, Mm00464608_m1, uPAR, and 18S rRNA (Hs99999901_s1; Thermo Fisher Scientific) were measured. The number of target genes was determined based on18S rRNA. A comparative threshold cycle (CT) was used to quantify the transcripts. The value was calculated using the 2^−ΔCT^ method.

### 2.4 PAI-1 stimulation

Approximately 5 × 10^5^ cells/well were seeded into a 24-well flat bottom plate and incubated overnight in 100 nM of human recombinant PAI-1 (rPAI-1, BioLegend, San Diego, CA, USA) with or without 100 μM TM5614 (a PAI-1 inhibitor). The cells were subsequently analyzed using flow cytometry. The supernatant from a subculture of the PAI-1-overexpressing cell line was collected after 2 d.

### 2.5 RNA extraction and real-time quantitative polymerase chain reaction

Total RNA was extracted from cells after 24 h of PAI-1 stimulation using ISOGEN II reagent (Nippon Gene, Tokyo, Japan). The RNA (0.5 μg) was reverse-transcribed to cDNA using the PrimeScript™ RT-PCR kit (Takara, Shiga, Japan), and gene expression levels were measured by qPCR analysis with TaqMan Fast. Appropriate TaqMan primers/probes (Thermo Fisher Scientific) were used: mouse *Pd-l1* (Mm00452054_m1), human *PD-L1* (Hs01125301_m1), and 18S ribosomal RNA (Hs99999901_s1). The expression levels of the target genes were determined relative to endogenous 18S ribosomal RNA (Hs99999901_s1). A comparative threshold cycle (CT) was used to quantify transcripts. The value was calculated using the 2^−ΔCT^ method.

### 2.6 Enzyme-linked immunosorbent assays

Murine or human tumor cells (2.5 × 10^4^ cell/well) were cultured for 4 days in 24-well plates with or without rPAI-1 stimulation (100 nM). Soluble PD-L1 in culture supernatants was measured using the mouse PD-L1/B7-H1 DuoSet ELISA kit (DY1-19; R&D System, Northeast, MN, USA) or human PD-L1 SimpleStep ELISA kit (ab277712; Abcam, Cambridge, UK) according to the manufacturer’s protocol. The detection ranges of the ELISA kits for murine and human soluble PD-L1 were 18.75–1200 pg/mL and 7.81–500 pg/mL, respectively.

### 2.7 Animal studies

All animal-related experimental procedures and protocols were designed according to the PREPARE guidelines (28) and reviewed and approved by the Institutional Animal Care and Use Committee at Tokai University (approval number: 231102). Animal handling and experimental procedures were performed in compliance with the ARRIVE guidelines 2.0 (29) and other relevant guidelines and regulations.

Six- to twelve-week-old female C57BL/6NJ, C3H/HeJ, and BALB/c nude (BALB/cAJc1-nu/nu) mice were obtained from CLEA (Tokyo, Japan). Rag2/IL2Rg-DKO (C;129S4-Rag2tm1.1Flv Il2rgtm1.1Flv/J) mice were acquired from Jackson Laboratory (Bar Harbor, ME, USA). The mice were housed under controlled humidity (50 ± 10%) and temperature (23 ± 2 °C) conditions with a standard 12 h light/dark cycle and provided *ad libitum* access to sterile water and a pellet diet in a barrier and specific-pathogen-free environment. The mice were randomly assigned to experimental groups without blinding the researchers, and no animals were excluded from analyses.

Between 5 × 10^5^ and 2 × 10^6^ tumor cells were subcutaneously injected into the right flank of the mice, and tumor volumes were calculated using a formula based on width (W) and length (L) measurements as follows:


$$\mathrm{Tumor}\;\mathrm{volume}\;=\;\left(L\;\times \;W^{2}\right)/2$$

After 7 d of cell inoculation, the tumor-bearing mice received TM5614 orally at a dose of 10 mg/kg daily for 14 days, either alone or in conjunction with an intraperitoneal injection of anti-PD-1 antibody or control IgG antibody (150 μg/mouse, Bio X Cell, Lebanon, NH, USA) thrice during the 14-day treatment period. Tumor samples were collected for further analysis. In another experiment, 5 × 10^5^ 32Dp210 leukemia cells were transplanted intravenously into C3H/HeJ mice. Seven days after cell inoculation, mice with tumors were orally administered vehicle or TM5614 (10 mg/kg) for 7 consecutive days. The day following the last administration, leukemia-infiltrated spleens were collected.

All mice were euthanized via cervical dislocation under full anesthesia induced by inhaled isoflurane (2.0% in air during anesthesia) by trained personnel in accordance with the American Veterinary Medical Association (AVMA) guidelines for the euthanasia of animals (2020).

### 2.8 T-cell isolation, activation, and co-culture

The murine spleens were minced into a homogenous paste using round-ended tweezers in phosphate-buffered saline (PBS) containing 5 mM EDTA and 2% bovine serum albumin (hereafter referred to as PEB buffer). Subsequently, cell suspensions were passed through a 40 μm nylon cell strainer (Corning, Corning, NY, USA) and incubated for 5 min with ammonium-chloride-potassium lysis buffer to lyse the red blood cells. Splenocytes were suspended in PEB buffer before negative selection with biotin-conjugated antibodies against CD11b, CD11c, CD19, CD45R (B220), CD49b, CD105, MHC class II, and Ter-199 to enrich T-cells.

T-cell isolation involved depleting magnetically labeled cells following the manufacturer’s guidelines (Miltenyi Biotec, Bergisch Gladbach, Germany). T cells were seeded in RPMI-1640 medium supplemented with human IL-2 (50 U/mL, Thermo Fisher Scientific), 2-mercaptoethanol (50 μM), and sodium pyruvate (1 mM) and activated using MACSiBeads particles, including biotinylated antibodies against CD3e and CD28, as per the manufacturer’s instructions. A total of 1 × 10^5^ MC38 or B16F10 cells were preincubated with 100 nM rPAI-1 in the presence or absence of 10 μg/mL anti-PD-L1 blocking antibody (BioLegend) and co-cultured overnight with 1 × 10^5^ T cells. BD Golgi Stop™ (BD Bioscience, Franklin Lakes, NJ, USA) was added during the last 4 h of co-culture to prevent perforin and granzyme B secretion.

### 2.9 T-cell-mediated cytotoxicity assay

MC38 cells (5 × 10^5^) were injected subcutaneously into the right flank of mice and left to establish for one week with no further intervention. Then, splenic CD8^+^ T cells were isolated from tumor-bearing mice via magnetic cell sorting with negative selection (CD8a^+^ T cell isolation kit, Miltenyi Biotec). CD8^+^ T cells were activated using MACSiBead particles loaded with monoclonal anti-CD3e and CD28 antibodies (Miltenyi Biotec) in RPMI-1640 medium supplemented with human recombinant IL-2 (1000 U/mL), 2-mercaptoethanol (50 μM), and sodium pyruvate (1 mM) and cultured for 2 days. MC38 cells were preincubated with 100 nM rPAI-1 overnight and stained with PKH67 (Sigma Aldrich). Subsequently, 1 × 10^4^ MC38 cells (target) were co-cultured with the activated CD8^+^ T cells (effector) at E:T ratios of 6.13:1 to 50:1 for 4 h. The PKH67^+^ MC38 cells were stained with PE/Cy7-conjugated anti-Annexin V antibody (BioLegend) and analyzed via flow cytometry. The cytotoxicity proportion was calculated as follows:


Cytotoxicity = [(ET – T0)/(100 – T0)] × 100%

where ET represents the percentage of PKH67^+^ Annexin V^+^ cells during the effector and target cell co-culture and T0 represents the percentage of PKH^+^ Annexin V^+^ cells in the target cell alone culture.

### 2.10 Flow cytometry

Flow cytometry was performed on a FACS LSRFortessa instrument using the FACSDiva software (BD Biosciences). Data analysis was performed using FlowJo^®^ software (Tree Star, Ashland, OR, USA). The proportion of the designated cell fraction was determined by collecting 100,000 events while excluding dead cells stained with propidium iodide from the data collection.

To analyze the expression of PD-L1, cells were stained with APC-conjugated anti-mouse PD-L1 (CD274, BioLegend). The following antibodies were used to identify the macrophages: APC-conjugated anti-mouse/human CD11b, PE-conjugated anti-mouse CD206 (BioLegend), PE-conjugated CD45, and AlexaFluor700-conjugated anti-mouse F4/80. Additionally, T cell activation was assessed using PE-conjugated CD3, FITC-conjugated CD8, and AlexaFluor700-conjugated CD69 antibodies. To identify regulatory T cells, PE-conjugated anti-mouse CD45, FITC-conjugated anti-mouse CD3, PE-conjugated anti-mouse CD25, and PerCP/Cy5.5-conjugated anti-foxp3 (BioLegend) antibodies were used. Cytofix/Cytoperm buffer (BD Biosciences) was used for the staining of intracellular proteins according to the manufacturer’s instructions. The following antibodies were used to detect the expression of signaling molecules: rabbit anti-PAI-1 (Abcam), rabbit anti-phospho-JAK1, rabbit anti-phospho-TYK2, and rabbit anti-phospho-STAT3 (Cell Signaling Technology, Danvers, MA, USA). Subsequently, a PE anti-rabbit secondary antibody was used. Finally, cytotoxic T cells were detected in tumor tissues using APC-conjugated anti-perforin and PE/Cy7-conjugated anti-granzyme B antibodies (BioLegend). Corresponding isotype-matched antibodies were used to determine the baseline staining for the analyses.

### 2.11 Immunohistochemistry

The hearts of isoflurane-anesthetized mice were perfused with 4% paraformaldehyde in PBS through the left ventricle. The tumors were excised, fixed in 4% paraformaldehyde, and embedded in paraffin. Deparaffinized sections were incubated with a rabbit anti-mouse α-smooth muscle actin (SMA) monoclonal antibody (Sigma-Aldrich) and visualized using a catalyzed signal amplification II system (Dako, Santa Clara, CA, USA). The slides were developed using DAB (diaminobenzidine) and counterstained with methyl green. For fluorescence immunohistochemistry, MC38 cells were cultured in a 4-well chamber slide (Thermo Fisher Scientific). The cells were then incubated with the primary antibody rabbit anti-phospho-Stat3 (Tyr705) (Cell Signaling Technology) and the secondary antibody Alexa Fluor 594 goat anti-rabbit IgG (Thermo Fisher Scientific), followed by counterstaining with DAPI (4′,6-diamidino-2-phenylindole). Images were obtained using an HS All-in-one Fluorescence Microscope Biorevo 9000 (Keyence, Osaka, Japan) and analyzed using BZ II analyzer software (Keyence).

### 2.12 Statistics and reproducibility

Data from a minimum of three independent experiments were combined and analyzed using GraphPad Prism version 10.0 (GraphPad Software, La Jolla, CA, USA). Results were expressed as means ± standard deviation (SD) of three to five independent experiments, with n values indicating the number of mice or samples per group. The Mann–Whitney unpaired *t*-test was used to compare the two groups. One-way analysis of variance was conducted, followed by Tukey’s *post-hoc* test, to compare the mean values among three or more independent groups. Pearson linear correlation analysis was performed to assess correlations between the mean fluorescent intensity (MFI) of PAI-1 and PD-L1 in tumor cells. The normality of the data was assessed using the Kolmogorov–Smirnov test. The level of statistical significance was set at *p* < 0.05.

## 3 Results

### 3.1 PAI-1 induces PD-L1 expression in various murine tumor cell types

**Figure 1:**
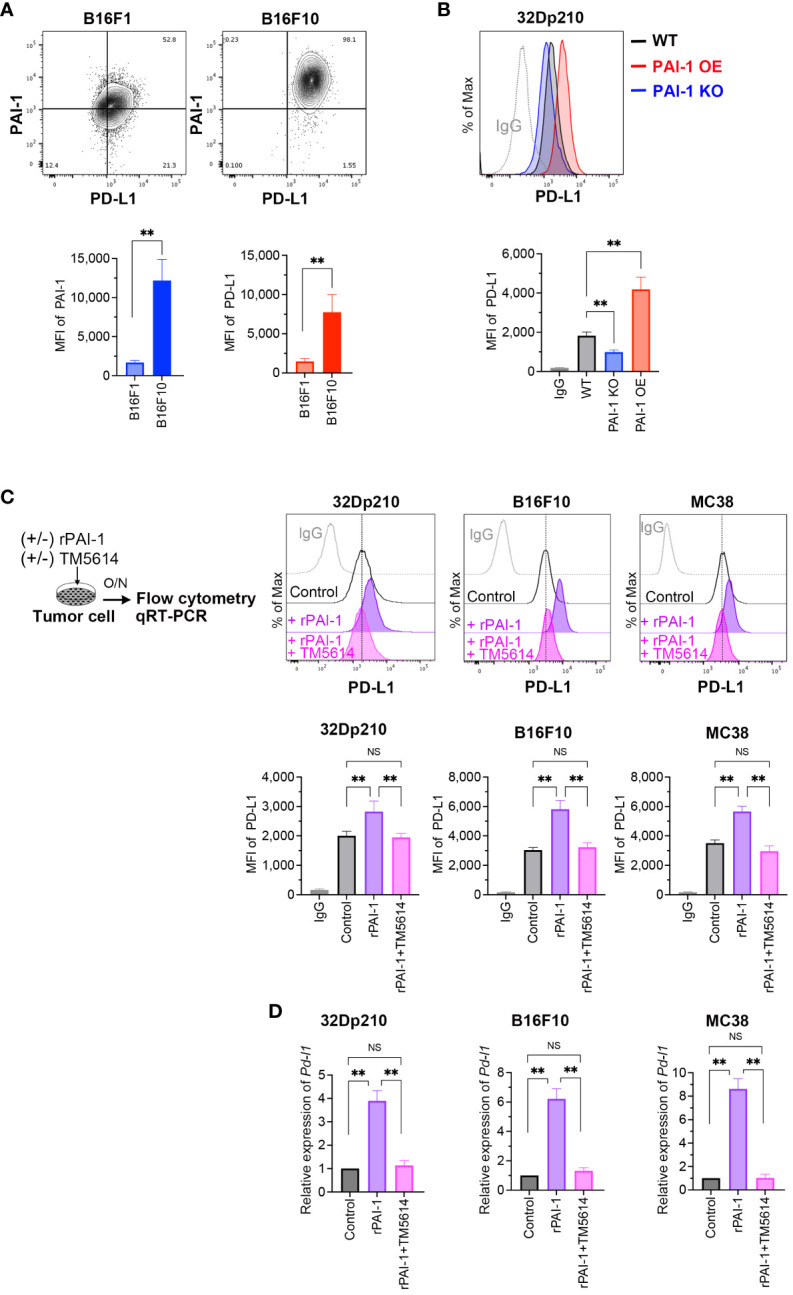
PAI-1 induces the expression of PD-L1 in mouse tumor cells. **(A)** Representative flow cytometry profiles and mean fluorescence intensity (MFI) (*n* = 6/group) demonstrating PAI-1 and PD-L1 expression in B16F1 and B16F10 melanoma cells. **(B)** Representative flow cytometry profiles and MFI (*n* = 6/cell type) showing PD-L1 expression in 32Dp210-WT, 32Dp210-PAI-1 OE, and 32Dp210-PAI-1 KO myeloid leukemia cells. **(C)** Representative flow cytometric profiles of PD-L1 expression and MFI (*n* = 6/group) in 32Dp210, B16F10, and MC38 cells incubated with 100 nM rPAI-1 with or without 100 μM of the PAI-1 inhibitor TM5614. **(D)** Relative expression of *Pd-l1* mRNA (*n* = 5/group) in 32Dp210, B16F10, and MC38 cells incubated with 100 nM rPAI-1 with or without 100 μM PAI-1 inhibitor, TM5614. Bars are expressed as means ± SD of three to five independent experiments. **p < 0.01, NS, non-significant.

A robust correlation between tumor malignancy and PAI-1 expression has been previously demonstrated (30–32). To better understand the tumor cells highly expressing PAI-1, we analyzed B16F10 cells—a murine melanoma subline with high lung metastatic ability—and B16F1 cells—a subline with low metastatic ability. Melanoma cells were double-stained with anti-PAI-1 and anti-PD-L1 antibodies; flow cytometry revealed a correlation between PAI-1 and PD-L1 expression (**Figure 1A**). Thus, we hypothesized that PAI-1 inhibits the immune surveillance systems of tumor cells by facilitating the expression of PD-L1 in tumors and tumor-surrounding cells. To test this hypothesis, we assessed the effects of PAI-1 on the activation of PD-L1 in murine tumor cell lines. 32Dp210 cells were genetically modified to enhance (PAI-1 overexpression (OE)) or eliminate (PAI-1 knockout (KO)) PAI-1 expression. Our findings revealed that leukemia cells overexpressing PAI-1 exhibited greater PD-L1 expression than the control group (i.e., 32Dp210 cells transfected with the control mock vector) (**Figure 1B**).

Considering that PAI-1 can cause autocrine and/or paracrine responses in itself and surrounding cell populations (14), we investigated the effect of PAI-1 secreted from tumor cells on PD-L1 expression. Murine tumor cell lines were treated with recombinant PAI-1 (rPAI-1) or the culture supernatant of 32Dp210 PAI-1 OE cells and cultured for 1 d. Subsequent analysis of PD-L1 expression by flow cytometry and qPCR revealed that rPAI-1 treatment induced high surface (**Figure 1C**) and mRNA (**Figure 1D**) expression of PD-L1. Hence, tumor-cell-secreted PAI-1 can trigger PD-L1 expression in murine tumor cells at the translational and transcriptional levels.

Next, we evaluated the effects of a compound capable of inhibiting PAI-1, namely TM5614. The tumor cells we treated with TM5614 and rPAI-1 and cultured for 1 d. The addition of the PAI-1 inhibitor completely abolished the induction of PD-L1 expression by rPAI-1 (**Figures 1C, D**). Hence, PAI-1 inhibition hinders PD-L1 induction in a diverse array of murine tumor types, alleviating the associated immune suppression.

### 3.2 PAI-I induces PD-L1 expression in various human tumor cell types

**Figure 2:**
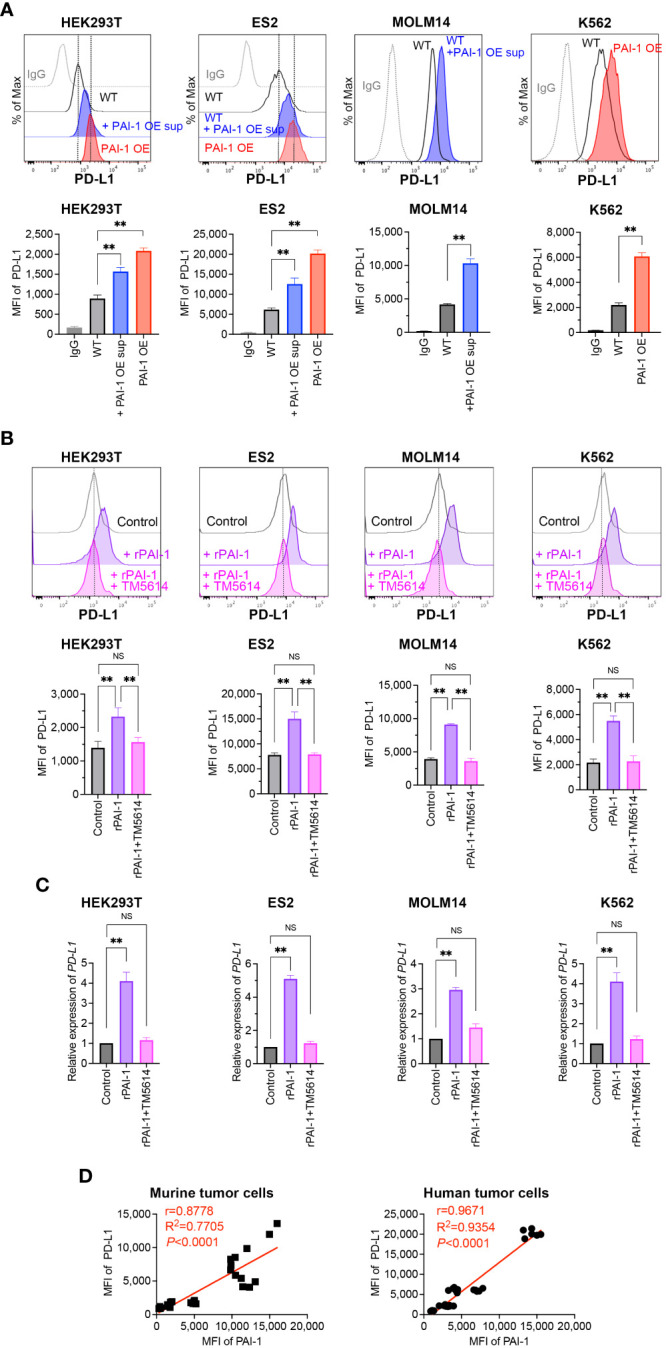
PAI-1 induces the expression of PD-L1 in human tumor cells. **(A)** Representative flow cytometric profiles of PD-L1 expression and MFI in the human cell lines HEK293T-WT, HEK293T-PAI-1 KO, ES2-WT, ES2-PAI-1 KO, MOLM14, K562-WT cells, and K562-PAI-1 OE cells treated with PAI-1 OE supernatant overnight. The experiments were repeated five times. **(B)** Representative flow cytometric profiles of PD-L1 expression and MFI (*n* = 5/group) in HEK293T, ES2, MOLM14, and K562 cells after incubated with 100 nM rPAI-1 with or without 100 μM PAI-1 inhibitor TM5614. **(C)** Relative expression of *PD-L1* mRNA in HEK293T, ES2, MOLM14, and K562 cells (*n* = 5/cell type) after incubation with 100 nM rPAI-1 with or without 100 μM of the PAI-1 inhibitor TM5614. **(D)** Pearson’s correlation analysis between MFI of PAI-1 and MFI of PD-L1 in murine tumor cell lines (B16F1, B16F10, 32Dp210-WT, and 32Dp210-PAI-1 OE) and human tumor cell lines (HEK293T-WT, HEK293T-PAI-1 OE, ES2-WT, ES2-PAI-1 OE, K562-WT, and K562-PAI-1 OE). *r:* Pearson’s correlation coefficient. Bars are expressed as means ± SD of three independent experiments. **p < 0.01, NS, non-significant.

To evaluate whether PAI-I also induces PD-L1 in human tumor cells, PAI-1-overexpressing (PAI-1 OE) HEK293T (fetal renal cells), ES2 (ovarian clear-cell adenocarcinoma cells), MOLM14 (acute myelogenous leukemia cells), and K562 (chronic myelogenous leukemia cells) cells were generated. Alternatively, the human tumor cells were treated with culture supernatants from PAI-1 OE cells (PAI-1 OE sup). A significant increase in PD-L1 expression levels was observed in all PAI-1 OE human tumor cell lines and tumor cells treated with the PAI-1 OE supernatant (**Figure 2A**).

The effect of rPAI-1 on human tumor cell lines was also assessed following treatment with or without a PAI-1 inhibitor (TM5614). After one day of culture, PD-L1 expression was analyzed via flow cytometry and qPCR. The addition of rPAI-1 induced high levels of PD-L1 surface and mRNA expression, which was abolished by the PAI-1 inhibitor (**Figures 2B, C**). Hence, PAI-1 induced PD-L1 expression in a range of human tumor types, including solid tumors and leukemia.

Given the apparent correlation between PAI-1 and PD-L1 expression (**Figures 1A**, **2A**), we assessed this trend held true for all mouse and human cell lines used in this study. The Pearson correlation analysis revealed a significant positive correlation between the expression of PAI-1 and PD-L1 (*p* < 0.0001, *r* > 0.8; **Figure 2D**) in murine and human tumor cell lines. This is consistent with the notion that cancers expressing high levels of PAI-1 are highly malignant.

### 3.3 PAI-1 promotes the production of soluble PD-L1

**Figure 3:**
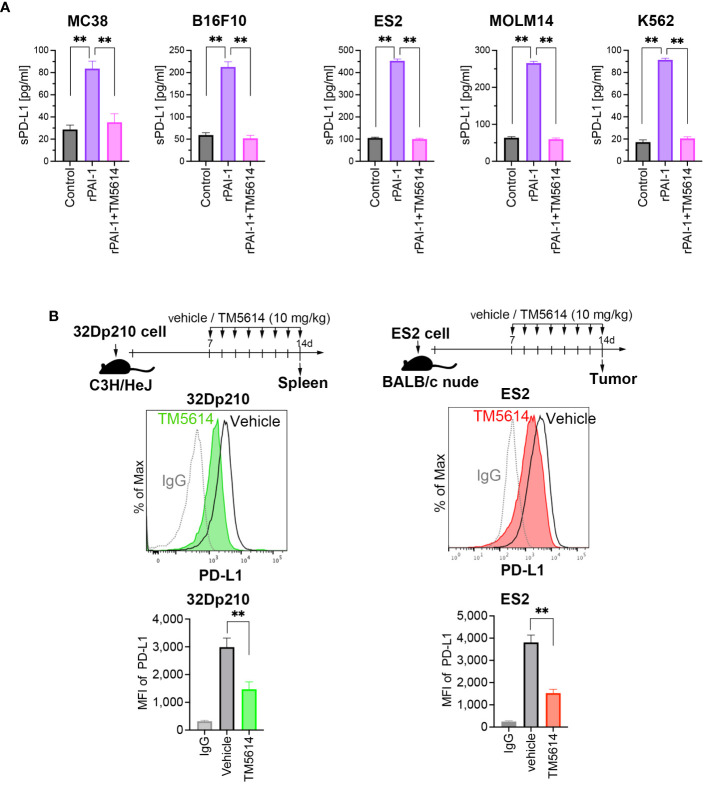
PAI-1 promotes the production of soluble PD-L1, whereas blockade of PAI-1 suppresses the expression of PD-L1 expression both *in vitro* and *in vivo.* **(A)** Production of soluble PD-L1 in culture supernatants from MC38, B16F10, ES2, MOLM14, and K562 cells (*n* = 5/cell type) after 4 days of incubation with 100 nM rPAI-1 with or without 100 μM of the PAI-1 inhibitor TM5614. **(B)** Representative flow cytometry graphs of PD-L1 expression and MFI were obtained from 32Dp210 cells engrafted in the spleen (*n* = 6) and ES2 cells (*n* = 6) engrafted in subcutaneous tissue at 14 days post-inoculation. Bars are expressed as means ± SD of three independent experiments. **p < 0.01.

Soluble and vesicle-associated PD-L1 reportedly exert a potent inhibitory effect on immune responses (33). Therefore, we sought to define whether the PAI-1-induced PDL-1 upregulation is associated with an increase in soluble PD-L1 or (and) vesicle-associated PDL-1. To this end, several murine and human tumor cell lines were cultured with rPAI-1 with or without TM5614. Four days after culture, the soluble PD-L1 in the culture supernatants was measured via ELISA. The data clearly demonstrate that PAI-1 induced the secretion of soluble PD-L1 in all the mouse or human tumor cell lines used in this study (**Figure 3A**). Therefore, we speculate that PAI-1-induced PD-L1 may exert a potent inhibitory effect on immune responses through both direct inhibition via cell–cell contact and indirect inhibition via soluble pathways.

### 3.4 PAI-1 inhibition downregulates PD-L1 expression *in vivo*

The efficacy of the PAI-1 inhibitor was evaluated *in vivo*. Transplantation of 32Dp210 cells into mice was performed intravenously, followed by oral administration of a PAI-1 inhibitor (TM5614) once daily at 10 mg/kg for 7 d. 32Dp210 cells engrafted in the spleen were harvested on the day following the final administration (Day 8). Additionally, ES2 cells were subcutaneously transplanted into nude mice. One week later, we confirmed the engraftment of a tumor mass > 5 mm along the major axis. Subsequently, the PAI-1 inhibitor was orally administered once daily at 10 mg/kg for 7 d. On the day after the final administration (Day 8), the tumor mass was harvested. As a control, physiological saline (vehicle) was administered to mice instead of TM5614.

The PD-L1 expression levels in the tumor cells were measured via flow cytometry. Mice that received the PAI-1 inhibitor had lower PD-L1 expression levels in murine and human tumor cells than the vehicle control mice (**Figure 3B**). These findings indicated that the PAI-1 inhibitor can also exert immunostimulatory effects *in vivo*.

### 3.5 PAI-1 induces PD-L1 expression in the tumor-surrounding environment

Our results suggest that the PAI-1 secreted from tumor cells may also induce PD-L1 expression in cell populations that constitute the tumor-surrounding environment, such as TAMs and CAFs, to evade immune cell attacks. Therefore, we analyzed the effects of PAI-1 on immunosuppressive TAMs (**Supplementary Figure S1**). We cultured murine splenocytes overnight, removed floating cells, and added rPAI-1 to adherent macrophages, which were then cultured for 4 d. Subsequently, the macrophages were collected and stained with anti-CD11b, anti-CD206, and anti-PD-L1 antibodies. We then gated CD11b and CD206 double-positive (CD11b^+^ CD206^+^) immunosuppressive macrophages and measured PD-L1 expression via flow cytometry.

PAI-1 stimulation did not affect the fluorescence intensity of CD11b or CD206, suggesting that PAI-1 has no effect on TAM differentiation (**Supplementary Figure S1B**). Meanwhile, the addition of rPAI-1 highly induced PD-L1 expression in TAMs compared to untreated control cells (**Supplementary Figure S1C**). However, treatment with the PAI-1 inhibitor suppressed the rPAI-1-induced enhancement of PD-L1 expression. These findings suggest that tumor cells secrete PAI-1, which induces PD-L1 expression in TAMs within the tumor-surrounding environment, contributing to immune escape.

### 3.6 PAI-1-induced PD-L1 suppresses T-cell-mediated cancer immunity

**Figure 4:**
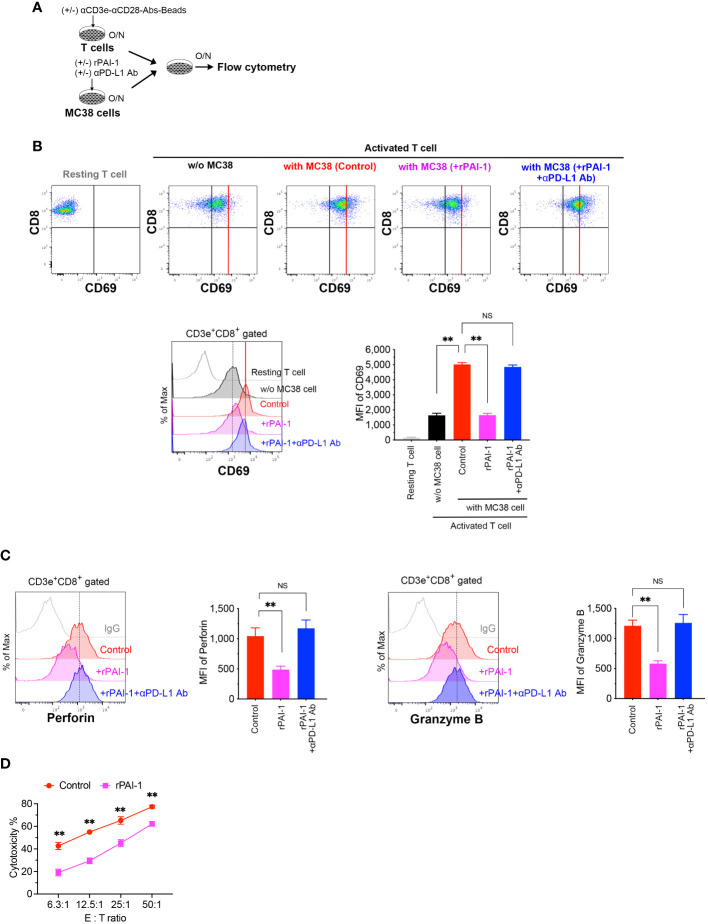
PAI-1-induced expression of PD-L1 in tumor cells impedes the activation of T-cells. **(A)** Experimental design: T cells were activated overnight with MACSiBead particles conjugated with monoclonal anti-CD3e and CD28 antibodies in a culture medium supplemented with IL-2. MC38 cells were pre-cultured overnight with or without 100 nM rPAI-1. A group was also prepared in which the anti-PD-L1 blocking antibody (10 μg/mL) was added during PAI-1 pre-stimulation. The T-cells and MC38 cells (1 × 10^5^ cells each) were subsequently mixed and co-cultured overnight. **(B)** Representative flow cytometric profiles and MFI for CD69 expression in CD3^+^ CD8^+^-gated resting or activated cytotoxic T cells, both before and after co-culturing with MC38. Each group contained six samples. **(C)** Representative flow cytometric profiles and MFI for the expression of perforin and granzyme B in CD3^+^ CD8^+^ cytotoxic T cells after co-culturing with MC38, with six samples in each group. **(D)** Cytotoxic activity of CD8^+^ T cells against MC38 cells after pre-incubation with or without rPAI-1 (*n* = 5/group). Bars are expressed as means ± SD of three independent experiments. ** *p* < 0.01, NS, non-significant.

To assess whether PAI-1-induced PD-L1 expression suppresses T-cell-mediated cancer immunity, T-cells were isolated from splenocytes and stimulated overnight with anti-CD3 and anti-CD28 antibodies. Simultaneously, rPAI-1 was added to the culture media of MC38 (**Figure 4A**) and B16F10 (**Supplementary Figure S2A**) cells to induce PD-L1 expression overnight. Subsequently, the T-cells and tumor cells were co-cultured overnight. The cells were collected and analyzed via flow cytometry after staining with anti-CD3, anti-CD8, anti-CD69, anti-perforin, and anti-granzyme B antibodies. The degree of T-cell activation was assessed by measuring CD69 expression—an antigen that is rapidly upregulated upon T-cell activation. As illustrated in **Figure 4B**; **Supplementary Figure S2B**, co-culturing CD8^+^ cytotoxic T-lymphocytes (CTLs) with tumor cells resulted in their activation, as evidenced by increased CD69 expression. However, in co-cultures where PAI-1 was added to tumor cells, no concomitant elevation in CD69 expression was observed. Similar trends were observed for the abundance of perforin and granzyme B (**Figure 4C**; **Supplementary Figure S2C**).

To further confirm that the reduced T cell activation caused by rPAI-1 was due to tumor-associated PD-L1, we added a PD-L1 blocking antibody to the co-culture assay. Neutralizing PD-L1 increased CD69, perforin, and granzyme B expression in CD8^+^ T cells. This suggests that the immunosuppression induced by PAI-1 was due to its effect on PD-L1 (**Figures 4B, C**).

Finally, we evaluated whether PAI-1-induced PD-L1 expression inhibited the cytotoxic activity of CTLs against tumor cells. To induce tumor-specific CTLs, MC38 cells were transplanted into mice. One week after transplantation, the spleens of tumor-bearing mice were removed and CD8^+^ T cells were isolated. The cells were then stimulated with anti-CD3 and anti-CD28 antibodies for 3 days. Simultaneously, MC38 cells were treated with rPAI-1 overnight to induce PD-L1 expression. CD8^+^ T cells were then co-cultured with MC38 cells and analyzed via flow cytometry. PAI-1-treated tumor cells exhibited apparent protection against CTL killing (**Figure 4D**; **Supplementary Figure S2D**). These findings further suggest that PAI-1-induced PD-L1 inhibits the T-cell targeting of tumor cells.

### 3.7 The PAI-1/LRP1/uPA/uPAR complex activates the JAK/STAT pathway to induce PD-L1 expression

**Figure 5:**
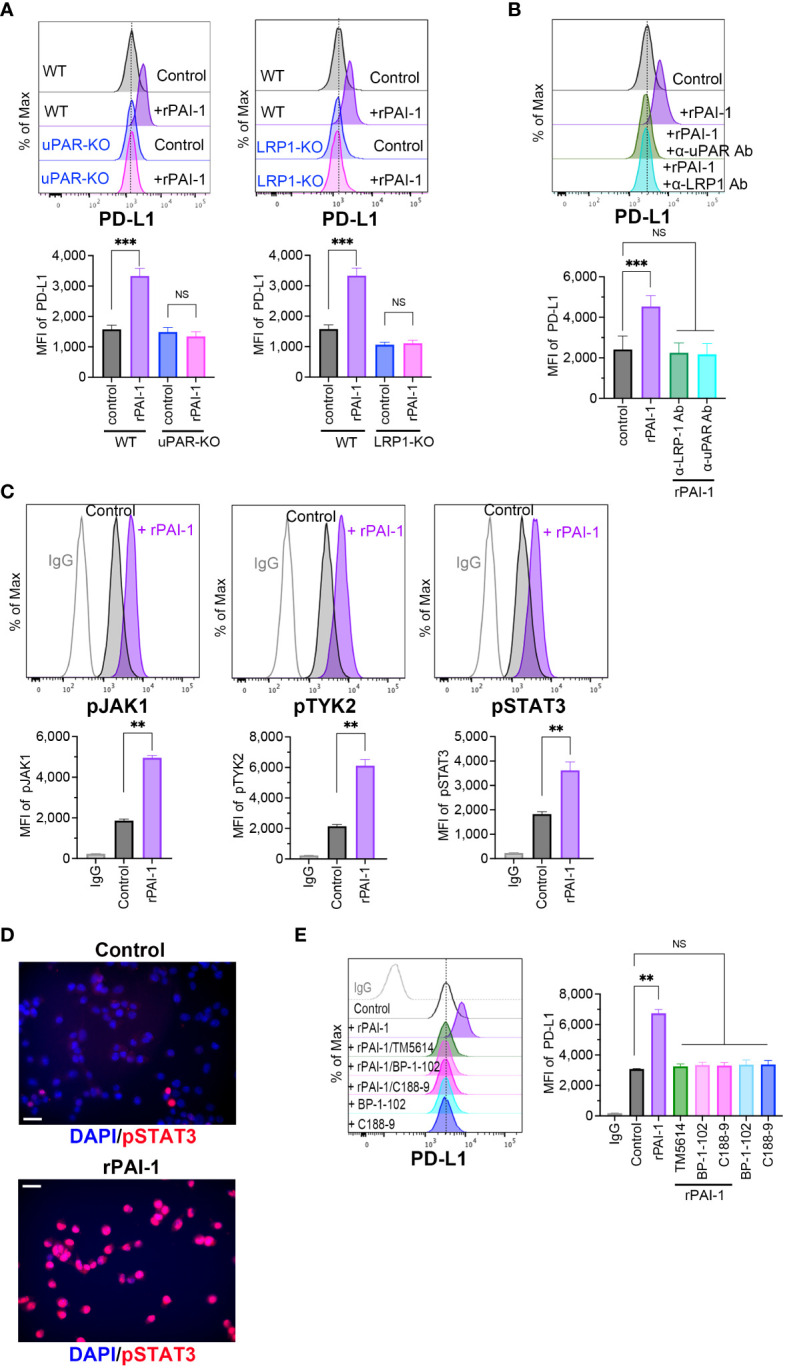
PAI-1-mediated induction of PD-L1 depends on the JAK/STAT signaling pathway induced by the PAI-1-uPAR-LRP1 complex. **(A)** Flow cytometric profiles show representative PD-L1 expression and MFI (*n* = 5/group) in wild-type (WT), uPAR-knockout (uPAR-KO), or LRP1-knockout (LRP1-KO) MC38 cells treated with or without 100 nM rPAI-1 overnight. **(B)** Flow cytometric profiles show representative PD-L1 expression and MFI (*n* = 5/group) in WT MC38 cells treated with 100 nM rPAI-1 and 100 ng/mL anti-uPAR Ab or anti-LPR-1 neutralizing antibody overnight. **(C)** Representative flow cytometry profiles and MFI (*n* = 6/group) show the expression of phosphorylated JAK1, TYK2, and STAT3 in MC38 cells after incubation with 100 nM rPAI-1 overnight. **(D)** Representative immunohistochemistry images depict phosphorylated STAT3 localization in MC38 cells with or without rPAI-1 treatment. Bars represent 20 μm. All experiments were repeated five times. **(E)** Representative flow cytometric profiles and MFI (*n* = 6/group) show the level of PD-L1 expression in MC38 cells after incubating with rPAI-1 and STAT3 inhibitors (BP-1-102 or C188-9, 10 μM) overnight. Bars are expressed as means ± SD of three independent experiments. **p < 0.01, ***p < 0.001, NS, non-significant.

Bound PAI-1 interacts with LRP1 to form the PAI-1/LRP1/uPA/uPAR complex, which activates intracellular signaling pathways (34, 35). To elucidate the mechanisms underlying the induction of PD-L1 expression by PAI-1, we prepared tumor cell lines deficient in uPAR (uPAR-KO; **Supplementary Figure S3A**) or LRP-1 (LRP1-KO; **Supplementary Figure S3B**) using the CRISPR/Cas9 system. Flow cytometric analysis demonstrated that PD-L1 induction did not occur when rPAI-1 was added to the culture medium of uPAR-KO or LRP1-KO cells (**Figure 5A**). Additionally, administration of the uPAR-neutralizing antibody inhibited PD-L1 induction in wild-type tumor cells (**Figure 5B**). These findings suggested that PAI-1 formed a complex with LRP1, uPA, and uPAR to trigger PD-L1 expression.

The PAI-1/LRP1/uPA/uPAR complex activates the JAK/STAT pathway (24, 25, 36), which includes several critical transcriptional factors that regulate PD-L1 by binding to its promoter. Therefore, we evaluated the involvement of the JAK/STAT pathway in PAI-1-induced PD-L1 expression. Tumor cells were treated with rPAI-1 and collected after 6 h; the JAK/STAT signaling pathway was analyzed via flow cytometry. The results confirmed that PAI-1 increased JAK1, TYK2, and STAT3 phosphorylation (**Figure 5C**). Additionally, immunohistochemical staining revealed that PAI-1 induced nuclear translocation of phosphorylated STAT3 (**Figure 5D**). Meanwhile, STAT3 inhibitors (BP-1-102 and C188-9) abolished the increase in PD-L1 expression induced by rPAI-1 (**Figure 5E**). These findings suggested that activation of the JAK/STAT pathway was responsible for PAI-1-induced PD-L1 induction.

### 3.8 PAI-1 blockade enhances tumor immunity activation

Our findings suggest that PAI-1 plays a critical role in promoting cancer progression via immunosuppression. However, given that PAI-1 inhibition does not completely eliminate PD-L1 expression, we assessed the effects of combined treatment with a PAI-1 inhibitor and anti-PD1 antibody. In this way, the therapeutic efficacy of PAI-1 inhibitors combined with checkpoint immunotherapy was evaluated.

MC38 (an immune checkpoint inhibitor (ICI)-sensitive cell line) or B16F10 (an ICI treatment-resistant cell line) cells were subcutaneously transplanted into mice. One week later, > 5 mm tumor masses were detected and engrafted onto the major axis. The mice were classified into four groups: (1) untreated (vehicle) control, *n* = 15 mice; (2) single dose of anti-PD1 antibody (anti-PD-1 Ab), *n =* 18 mice; (3) single dose of PAI-1 inhibitor (PAI-1 inhibitor), *n* = 18 mice; (4) combined anti-PD1 antibody and PAI-1 inhibitor (anti-PD-1 Ab+PAI-1 inhibitor), *n* = 18 mice. The PAI-1 inhibitor was orally administered once daily to groups 3 and 4 for 14 consecutive days. For groups 2 and 4, the anti-PD1 Ab was administered intraperitoneally every 3 d for a total of three times, beginning on the same day as the PAI-1 inhibitor administration. Physiological saline was orally administered to the untreated group. The tumor mass size was measured at 2 and 3 weeks after transplantation.

**Figure 6:**
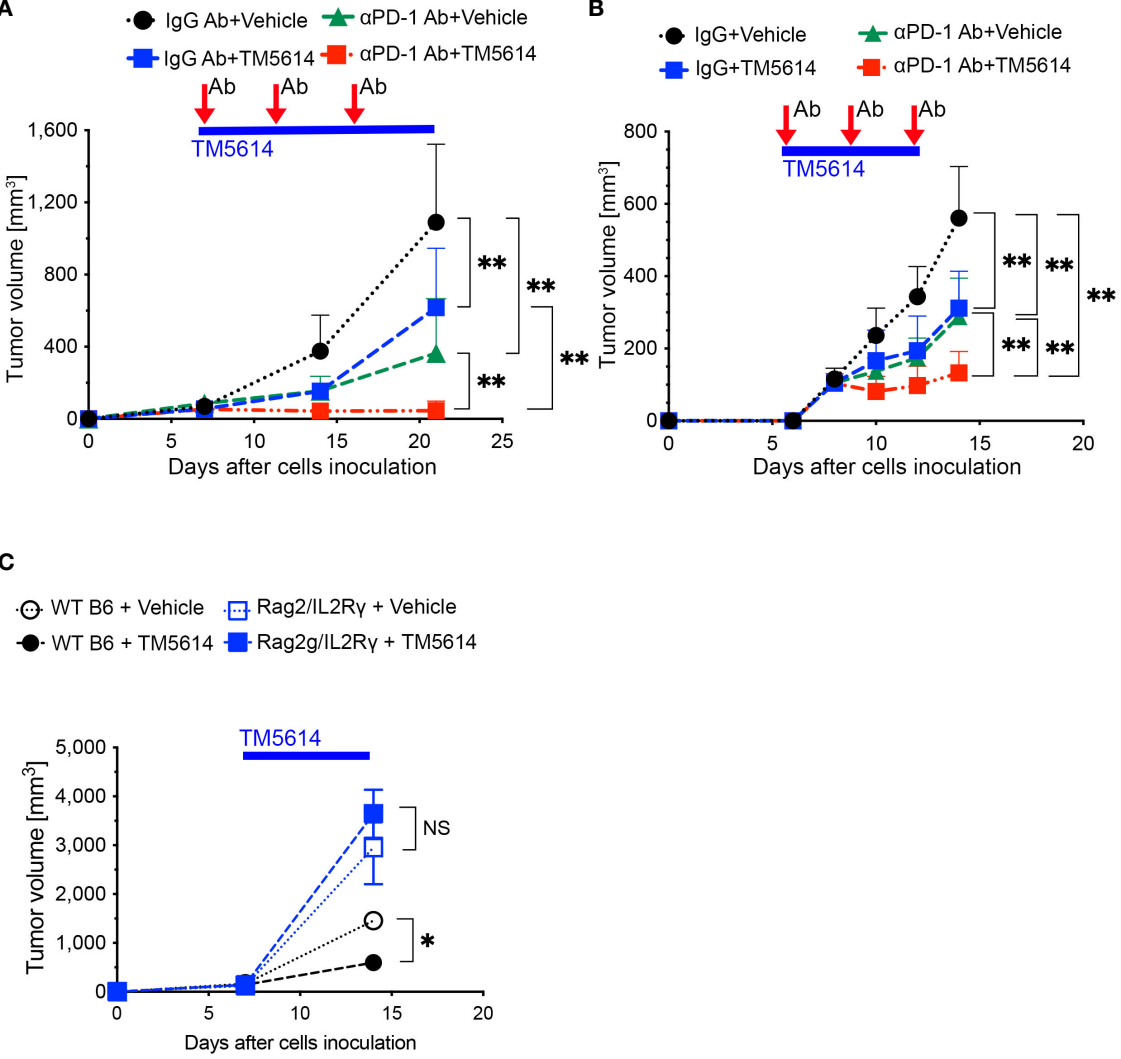
PAI-1-induced PD-L1 in tumor cells aids them in evading immune attack, and TM5614 synergizes PD-1 immuno-checkpoint therapy to suppress tumor growth. **(A)** Tumor volume in C57BL/6N mice (*n* = 15/group) after inoculation of 5 × 10^5^ MC38 cells. Precisely 150 μg of anti-PD-1 Ab or IgG Ab was intraperitoneally injected into each mouse (three shots), and the vehicle or TM5614 (10 mg/kg) was administered orally every day. **(B)** Tumor volume in C57BL/6N mice (*n* = 10/group) after inoculation of 5 × 10^5^ B16F10 cells. Anti-PD-1 Ab or IgG Ab was administered to the mice intraperitoneally (three shots), and the vehicle or TM5614 was administered orally every day. **(C)** Tumor volume in WT C57BL/6N mice (*n* = 10) or *Rag2/Il2rg-DKO* mice (*n* = 10) after inoculation of 5 × 10^5^ MC38 cells. The mice were orally administered the vehicle or 10 mg/kg of TM5614 every day. Plots are expressed as means ± SD of three independent experiments. **p < 0.01, NS, non-significant.

As shown in **Figures 6A, B**, the untreated group exhibited an increase in tumor diameter, whereas the single-administration anti-PD-1 Ab and PAI-1 inhibitor groups displayed tumor regression. Meanwhile, the anti-PD-1 Ab+PAI-1 inhibitor group exhibited complete disappearance of the tumor mass. These findings suggest that PAI-1 inhibitors and anti-PD-1 Abs have individual anti-tumor effects, but when used together, they exhibit enhanced anti-tumor effects.

To verify that the PAI-1 inhibitor activated tumor immunity by reducing PD-L1 expression, we transplanted MC38 cells subcutaneously into immunodeficient mice with a double *Rag2* and *Il2rg* (IL-2 receptor γ chain) knockout (Rag2/IL-2Rγ-double knockout, DKO) and no T, B, or NK cells; wild-type (WT) mice served as the control. After 1 week, each mouse was orally administered the PAI-1 inhibitor or physiological saline once daily for 14 consecutive days. The tumor regression effect of the PAI-1 inhibitor in WT mice was not observed in the immunodeficient mice (**Figure 6C**). These findings further suggest that the PAI-1 inhibitor promotes tumor regression by stimulating immunity.

### 3.9 PAI-1 inhibition ablates immunosuppression and enhances tumor responsiveness to immunotherapy

**Figure 7:**
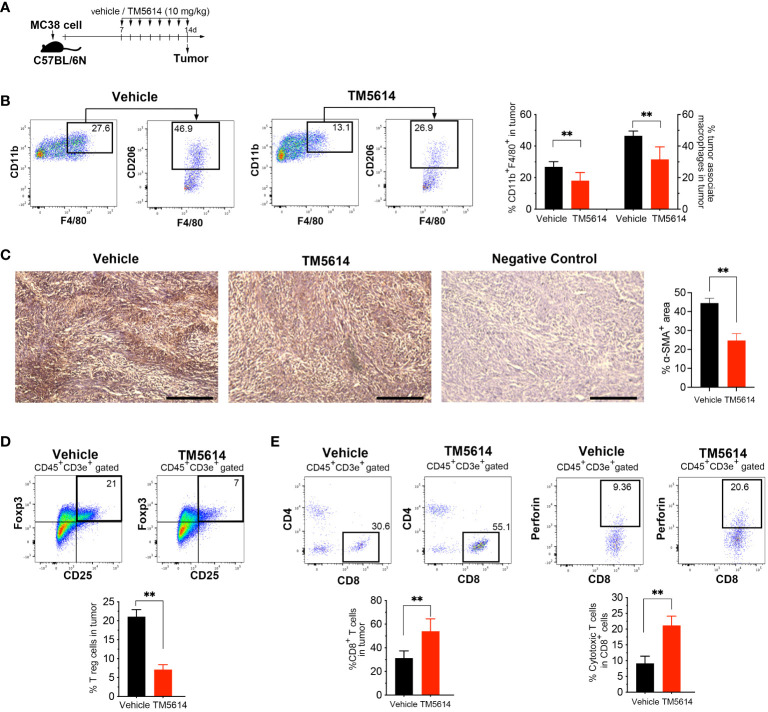
Pharmacological inhibition of PAI-1 activity restores tumor immunity. **(A)** Experimental design: Approximately 5 × 10^5^ MC38 cells were subcutaneously injected into the right flank of the mice. The mice were then orally administered TM5614 (10 mg/kg) or a control vehicle. Six animals were included in each group (three independent experiments combined). Tumors were harvested at 14 days post-inoculation. **(B)** Representative flow cytometric profiles along with the percentages of CD11b^+^F4/80^+^CD206^+^ tumor-associated macrophages in the tumor mass. **(C)** Representative images depicting α-SMA staining (brown staining) and the average percentage of area occupied by α-SMA-positive cancer-associated fibroblasts in the tumor mass. Bars represent 200 μm. **(D)** Representative flow cytometric profiles along with the percentages of regulatory T cells among the tumor mass. **(E)** Representative flow cytometric profiles along with the percentages of perforin^+^CD8^+^ cytotoxic T cells among the tumor mass. Bars are expressed as means ± SD of three independent experiments. **p < 0.01.

Malignant tumors contain a high proportion of immunosuppressive cell types, such as TAMs, CAFs, and regulatory T-cells (Tregs), as well as a dearth of effector T-cells (19, 20). Accordingly, we investigated whether the decreased cancer progression observed following treatment with a single PAI-1 inhibitor was associated with changes in the tumor immune cell infiltrate (**Figure 7A**). Staining for the macrophage markers CD45, CD11b, F4/80, and CD206 indicated a substantial decrease in TAM infiltration in the tumors of PAI-1-inhibitor-treated mice (**Supplementary Figure S4A**; **Figure 7B**). Moreover, immunohistochemical staining revealed decreased SMAα^+^ CAF infiltration in tumors following PAI-1 inhibitor treatment (**Figure 7C**).

Next, we evaluated the presence of Tregs by co-staining the CD4 and CD25 surface markers and the nuclear transcription factor FoxP3. Tumor cells treated with the PAI-1 inhibitor contained 60% fewer Tregs than did vehicle-treated tumor cells (**Supplementary Figure S4B**; **Figure 7D**). We also observed a two-fold increase in the number of CD8^+^ T-cells in PAI-1-inhibitor-treated mice, with a corresponding increase in perforin expression, suggesting functional CTLs (**Figure 7E**). Collectively, our findings strongly indicate that treatment with a PAI-1 inhibitor reduced immunosuppression and enhanced the anti-tumor CTL response.

## 4 Discussion

**Figure 8:**
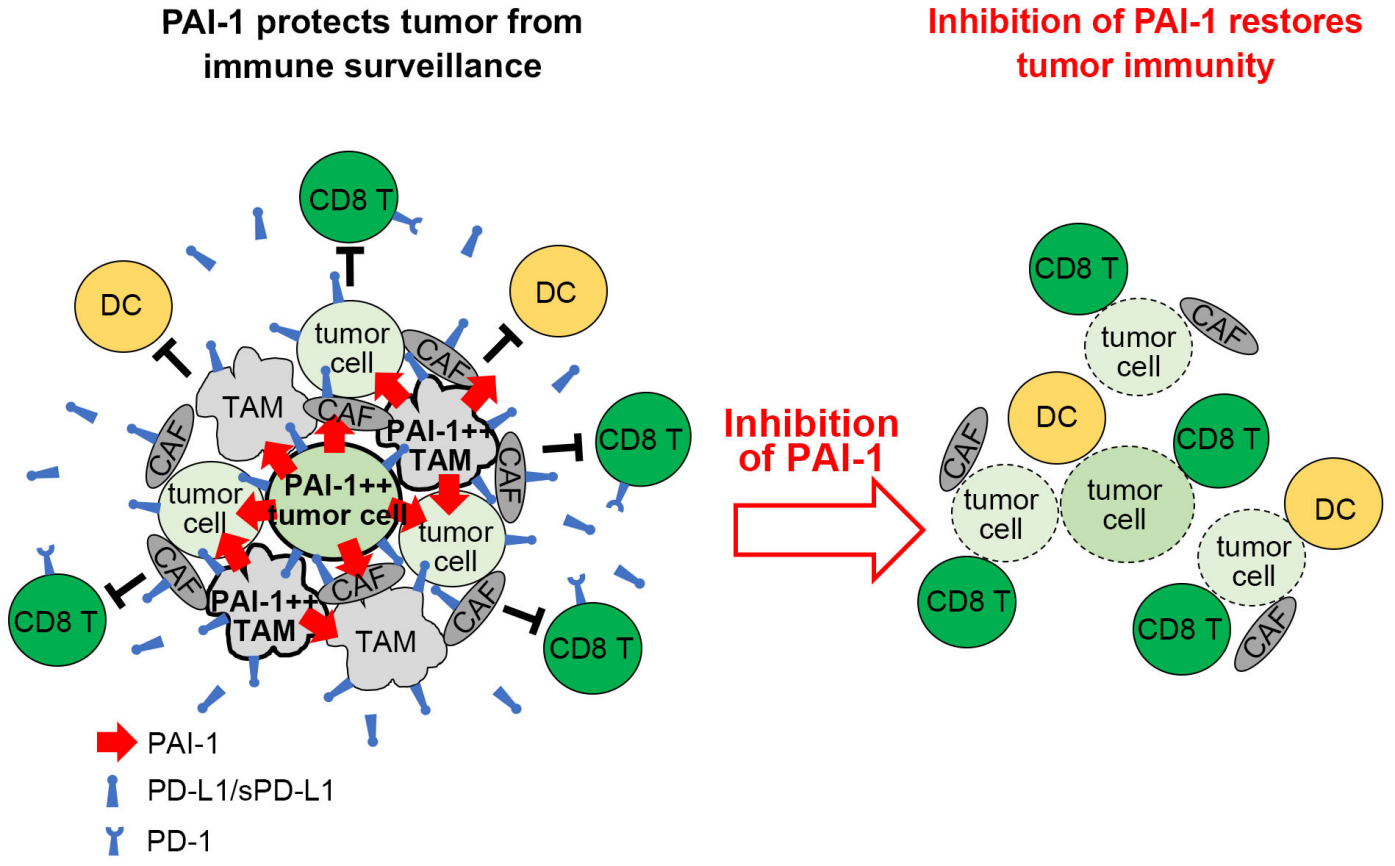
Graphical summary. Tumor cells expressing high levels of PAI-1 induce PD-L1 upregulation on themselves and surrounding cells, allowing them to escape immune surveillance. Inhibiting PAI-1 by TM5614 suppresses PD-L1 expression and enhances the efficacy of immune checkpoint inhibitor therapy.

It has been demonstrated that several types of tumor cells expressing elevated levels of PAI-1 paradoxically display a high degree of malignancy (6–8). This paradox is explained as follows. In addition to its fibrinolytic function, PAI-1 performs a wide range of pro-tumorigenic actions, including facilitating tumor vascularization, stimulating tumor progression and mobility, and inhibiting programmed cell death in tumors (11, 12, 37–39). This study further reveals that PAI-1 elicits pro-tumorigenic effects by suppressing the immune system (**Figure 8**). Mechanistically, the PAI-1 secreted by tumor cells induced the surface expression and secretion of PD-L1 by tumor cells via the LRP1/uPAR/JAK/STAT axis. Furthermore, tumor-cell-secreted PAI-1 upregulated PD-L1 in cell populations that make up the tumor-surrounding microenvironment, including TAMs and CAFs, to evade immune cell attack. This phenomenon enables tumor cells to bypass immune surveillance, increasing the likelihood of metastasis and other factors that increase the potential for poorer outcomes. The induction of PD-L1 by PAI-1 may be a fundamental principle observed in numerous tumor types, whether solid or hematopoietic in origin. PAI-1 inhibitors are expected to prevent the induction of PD-L1 by PAI-1, which occurs in a broad range of tumor types. This inhibition is anticipated to counteract the immunosuppression resulting from PD-L1 induction and inhibit the associated tumor metastasis. Therefore, PAI-1 inhibitors are expected to be useful immunostimulators in a wide range of tumor types.

PAI-1 serves as the primary inhibitor of uPA and tissue-type plasminogen activator (tPA), which transform plasminogen into plasmin (40). This system of plasmin activators and inhibitors is instrumental in regulating fibrinolysis and extracellular matrix remodeling, as well as tumor cell migration and invasion. PAI-1 participates in the regulation of tumor invasion and angiogenesis through interactions with binding partners such as LRP1 and uPAR (36–38). The PAI-1/LRP1/uPAR complex also stimulates the migration and polarization of monocytes and immunosuppressive macrophages in cancer (35). The complex interaction between these factors is responsible for malignancy progression. Our CRISPR/Cas9 and neutralizing antibody experiments revealed that PAI-1 facilitates PD-L1 expression via an LRP1/uPAR-dependent pathway. Further analysis of the signaling pathways indicates that the PAI-1/LRP1/uPAR complex enhances PD-L1 expression in a JAK/STAT3-pathway-dependent manner. This pathway includes various crucial transcription factors that regulate the transcriptional activation of PD-L1 (22). Indeed, PAI-1-induced STAT3 activation plays a vital role in promoting tumor progression by enabling pro-tumorigenic polarization of TAMs (35), inducing epithelial–mesenchymal transition in cancer (41) and promoting peritoneal carcinomatosis (42). Our findings provide new evidence that the activation of STAT3 by PAI-1 contributes to immune evasion, highlighting the PAI-1/STAT3 axis as a crucial target for therapeutic intervention.

PAI-1 was previously reported to downregulate PD-L1 based on the increased levels of PD-L1 expression in cells treated with siRNA to PAI-1 (23). However, our results consistently show a positive correlation between PAI-1 and PD-L1 expression. Interestingly, PAI-1 inhibitors were suppressive to tumor growth in both studies (23) (**Figures 6A, B**). This evidence of an increased anti-tumor immune response does not contradict our previous findings that PAI-1 inhibition reduced PD-L1 expression and rendered tumor cells vulnerable to immune attack. Transforming growth factor-β (TGF-β) and tumor necrosis factor-α (TNF-α) are potent inducers of PAI-1 and negative modulators of the immune response to tumor cells. TGF-β and TNF-α induce PD-L1 expression in tumors to promote immune evasion, while blockade of these factors can overcome resistance to ICI treatment (43–45). Therefore, TGF-β and/or TNF-α within the tumor microenvironment trigger PAI-1 expression, controlling the immune surveillance escape mechanism by increasing PD-L1 expression. Thus, a PAI-1 inhibitor as a single agent is useful as a PD-L1 expression inhibitor, immune stimulator, ICI of PD-L1, inhibitor of tumor cell exacerbation caused by PD-L1, and enhancer of tumor immunotherapy.

We have developed a synthetic small-molecule inhibitor of PAI-1, TM5275, and a series of its analogs with improved pharmacological and toxicological properties, including TM5509 and TM5614 (26, 46, 47). Several preclinical studies have demonstrated the ability of this series of compounds to affect various metabolic (48, 49), fibrotic (50), age-related (51, 52), and hematopoietic disorders (27, 53–55) and cancer (56, 57). This study reveals that PAI-1 inhibition may provide a therapeutic benefit in treating various tumors for which ICI therapy is applied. The PAI-1 inhibitor used in this study has completed phase I clinical trials with no significant adverse effects, including myelosuppression, observed. It has also shown efficacy in phase II clinical trials in patients with cancer and is currently in phase III trials. In patients diagnosed with melanoma, studies have reported positive and negative correlations between PAI-1 and PD-L1 expression (23, 58), but the clinical relevance of their relationship remains unclear. Indeed, this correlation may vary depending on disease progression and treatment duration. Hence, given the versatility of PAI-1 in cancer progression, including inhibiting apoptosis (10), controlling migration (27, 35), recruiting TAMs (59), and promoting fibrosis (60), further research is needed to comprehensively understand its influence on cancer progression. Nevertheless, combining our findings with previous research implicates PAI-1 as a crucial target for effective cancer therapy; hence, the selective blockade of PAI-1 using TM5614 and its derivatives may be of value for treating patients with various malignant tumors.

In conclusion, this study describes a novel role for PAI-1 in the immune evasion of tumor cells through the upregulation of PD-L1 expression, revealing its paradoxical pro-tumorigenic function. Furthermore, we partially elucidate the mechanism underlying the potential efficacy of combining PAI-1 inhibition and immunotherapy in the treatment of malignant tumors.

## Data availability statement

The original contributions presented in the study are included in the article/**Supplementary Material**. Further inquiries can be directed to the corresponding author.

## Ethics statement

Ethical approval was not required for the studies on humans in accordance with the local legislation and institutional requirements because only commercially available established cell lines were used. The animal study was approved by the Institutional Animal Care and Use Committee at Tokai University. The study was conducted in accordance with the local legislation and institutional requirements.

## Author contributions

AI: Formal analysis, Funding acquisition, Writing – original draft, Data curation, Investigation, Methodology, Visualization. TF: Formal Analysis, Investigation, Writing – review & editing. TU: Investigation, Writing – review & editing. TT: Investigation, Writing – review & editing. KH: Formal analysis, Investigation, Writing – review & editing. HH: Formal analysis, Investigation, Writing – review & editing. AO: Formal analysis, Resources, Writing – review & editing. TM: Resources, Supervision, Writing – review & editing. KA: Supervision, Writing – review & editing. TY: Conceptualization, Data curation, Formal analysis, Funding acquisition, Project administration, Supervision, Validation, Writing – original draft, Writing – review & editing, Investigation, Methodology, Resources, Visualization.
