# Supplementary material for: Plasminogen activator inhibitor-1 promotes immune evasion in tumors by facilitating the expression of programmed cell death-ligand 1
Source: Front Immunol. 2024 May 8;15:1365894. doi: 10.3389/fimmu.2024.1365894 (PMC11109370; doi:10.3389/fimmu.2024.1365894)
Supplement: Supplementary file 1 [file DataSheet_1.pdf]

Supplementary Materials

1 Supplementary Figures and Tables

1.1 Supplementary Figures

Supplementary Figure S1

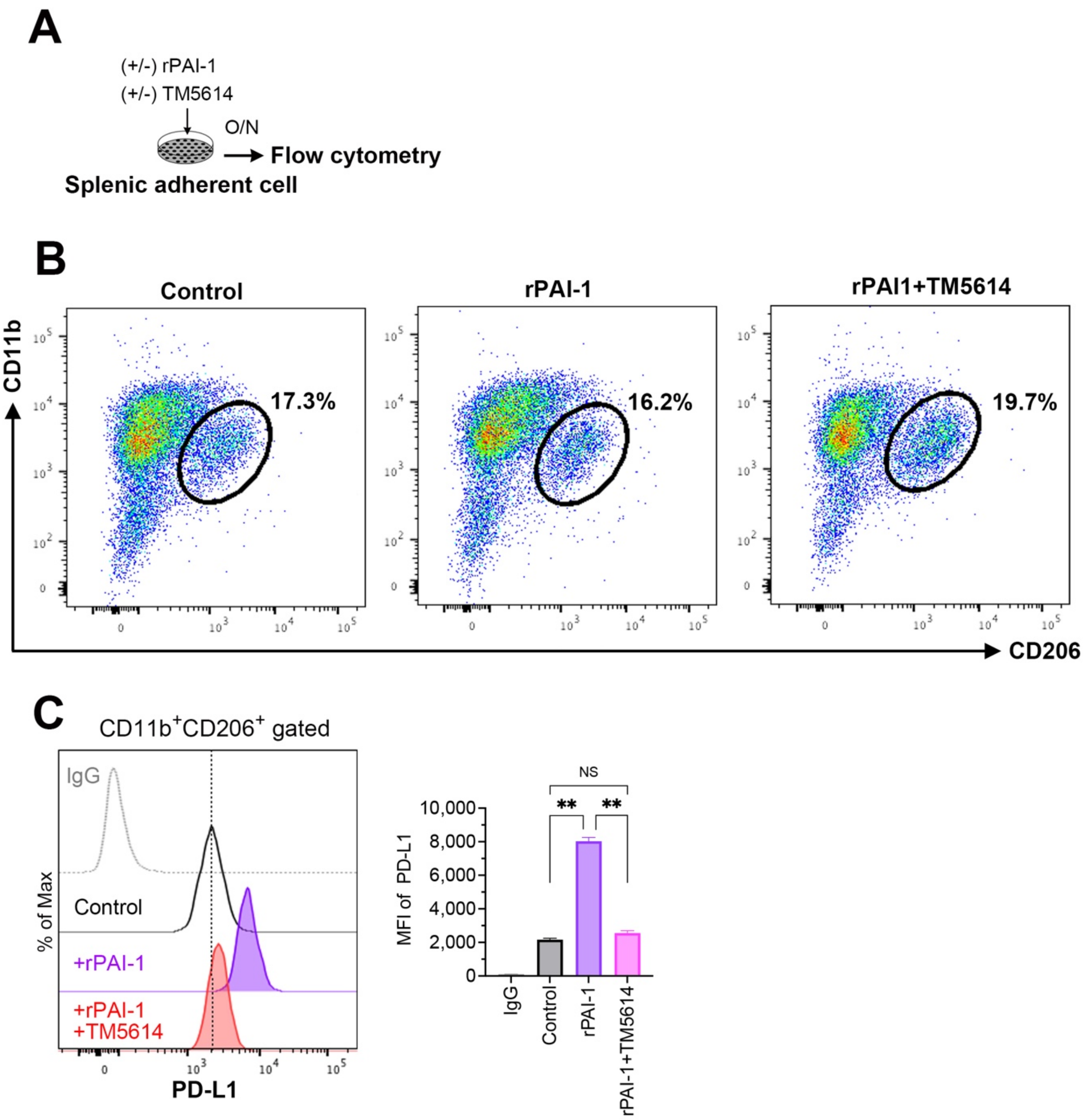

Supplementary Figure 1. PAI-1 induces PD-L1 expression in splenic macrophages. (**A**) Experimental design. Approximately 5 × 10^5 splenic mononuclear cells were cultured for 3 days in a medium supplemented with 10 ng/mL recombinant macrophage colony-stimulating factor (M-CSF). Splenic macrophages were stimulated with rPAI-1 with or without TM5614. (**B**) Representative flow cytometric profiles and (**C**) mean fluorescence intensity (MFI) (n = 6/group) depict the level of PD-L1 expression in macrophages treated with 100 nM rPA-1 overnight with or without 100 μM TM5614. **P < 0.01, NS: non-significance.

Supplementary Figure S2

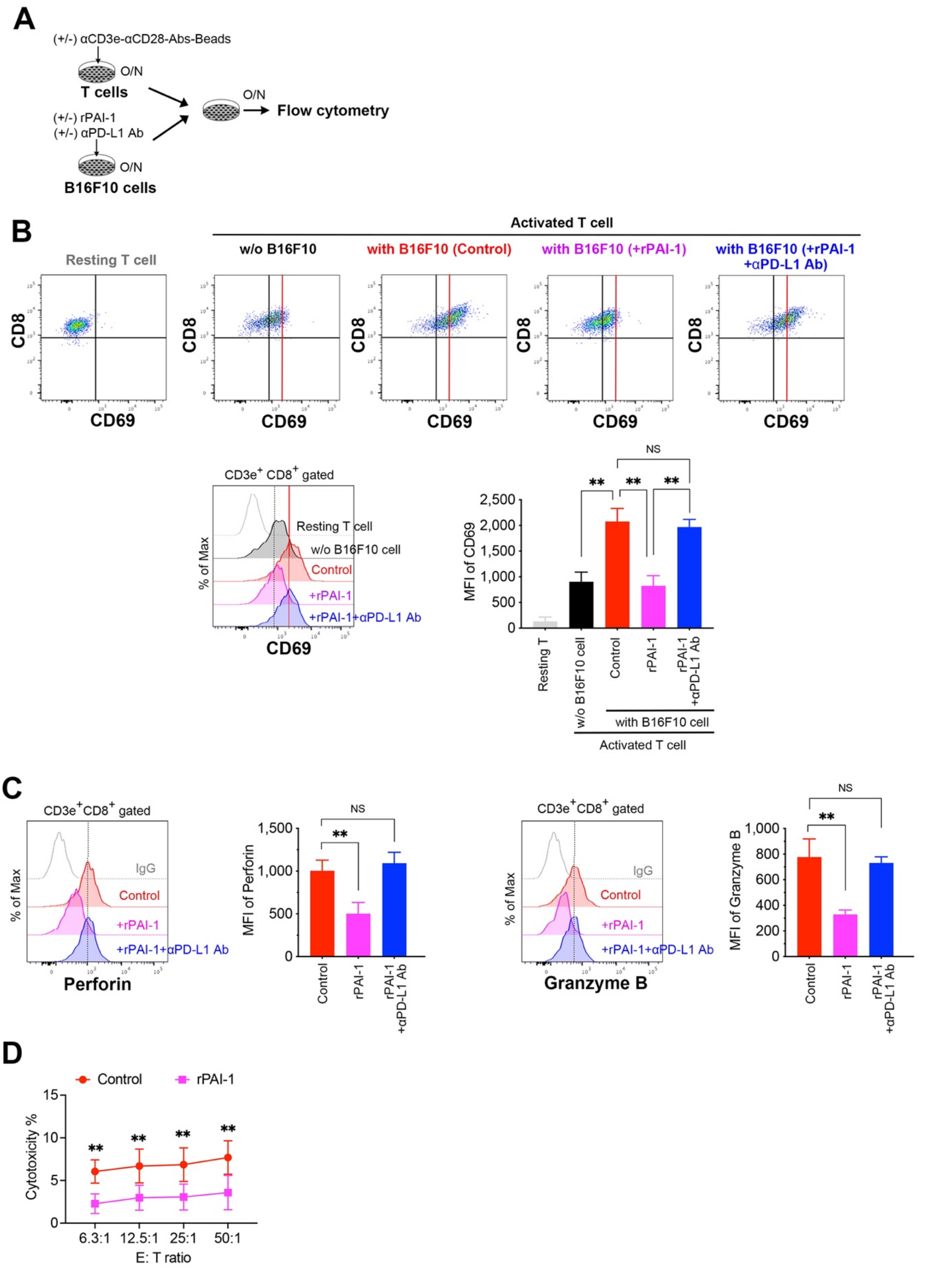

Supplementary Figure 2. PAI-1 induction of PD-L1 in B16F10 cells suppresses $CD8^+$ T-cell function. (**A**) Experimental scheme for T and B16F10 cell co-culture. T-cells from splenocytes were enriched, expanded, and activated using MACSiBeads particles conjugated with monoclonal CD3/CD28 antibodies. The culture medium was supplemented with IL-2 overnight. B16F10 cells were pre-cultured overnight with or without 100 nM rPAI-1. In one group, an anti-PD-L1 blocking antibody (10 μg/mL) was added during PAI-1 pre-stimulation. A total of 1×10^5 CD8 T-cells and 1×10^5 B16F10 cells were co-cultured overnight in 96-well plates. (**B**) Representative flow cytometric profiles, histograms, and mean fluorescence intensity (MFI) for CD69 regarding resting and B16F10-stimulated $CD8^+$ T-cells ($n = 6$) with and without rPAI-1 treatment. (**C**) Representative flow cytometric profiles and MFI for perforin and granzyme B produced by CD8 T-cells ($n = 6$) after co-culturing with B16F10 cells. (**D**) Cytotoxic activity of $CD8^+$ T cells against B16 cells after pre-incubation with or without rPAI-1 (n = 5/group). $^{**}P < 0.01$, NS: non-significance.

Supplementary Figure S3

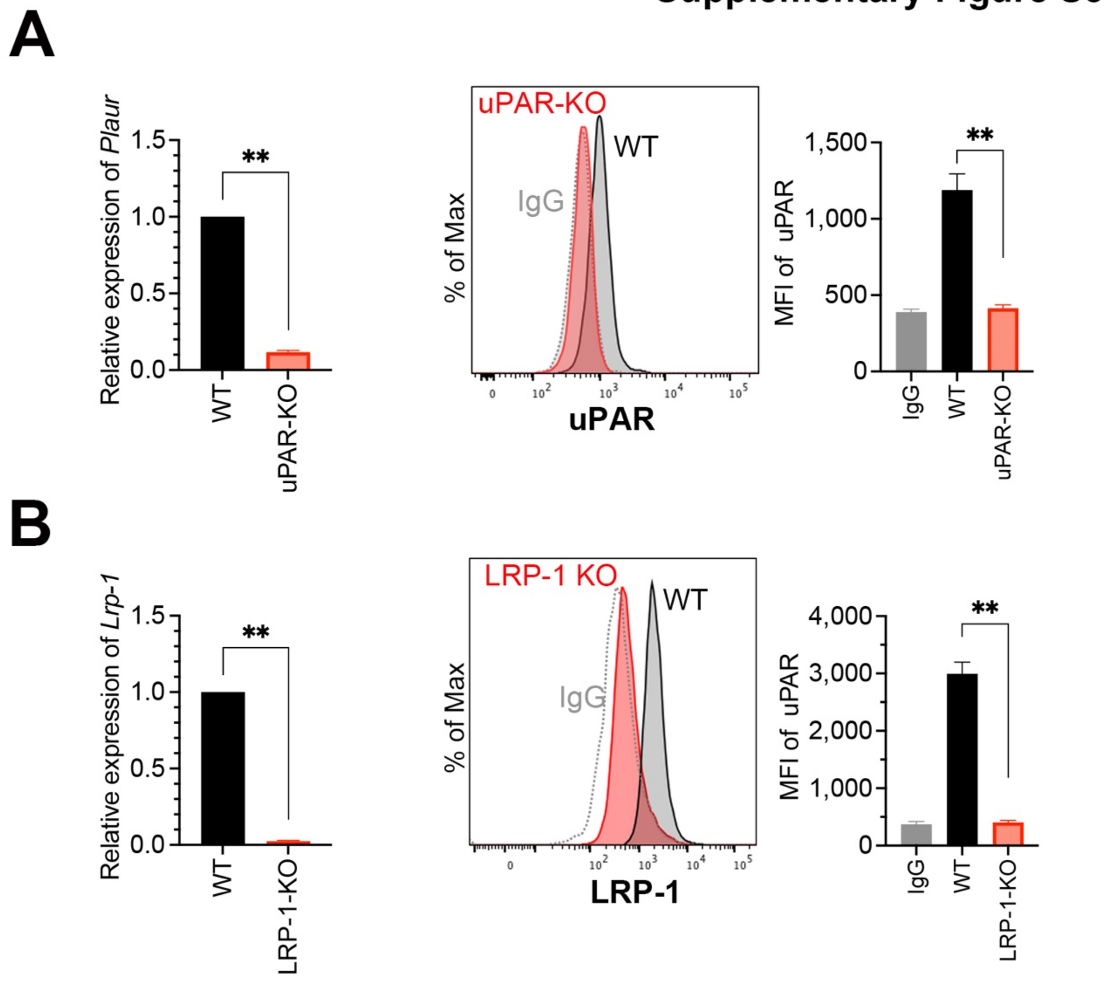

Supplementary Figure 3. Validation of gene-targeting in cells; related to Figure 5A. Relative mRNA expression of target genes; representative flow cytometric profiles and mean fluorescence intensity (MFI) ($n = 5$) for uPAR (*Plaur*) (**A**) or LRP1 (**B**) expression in gene-targeted cells. $^{**}P < 0.01$.

Supplementary Figure S4

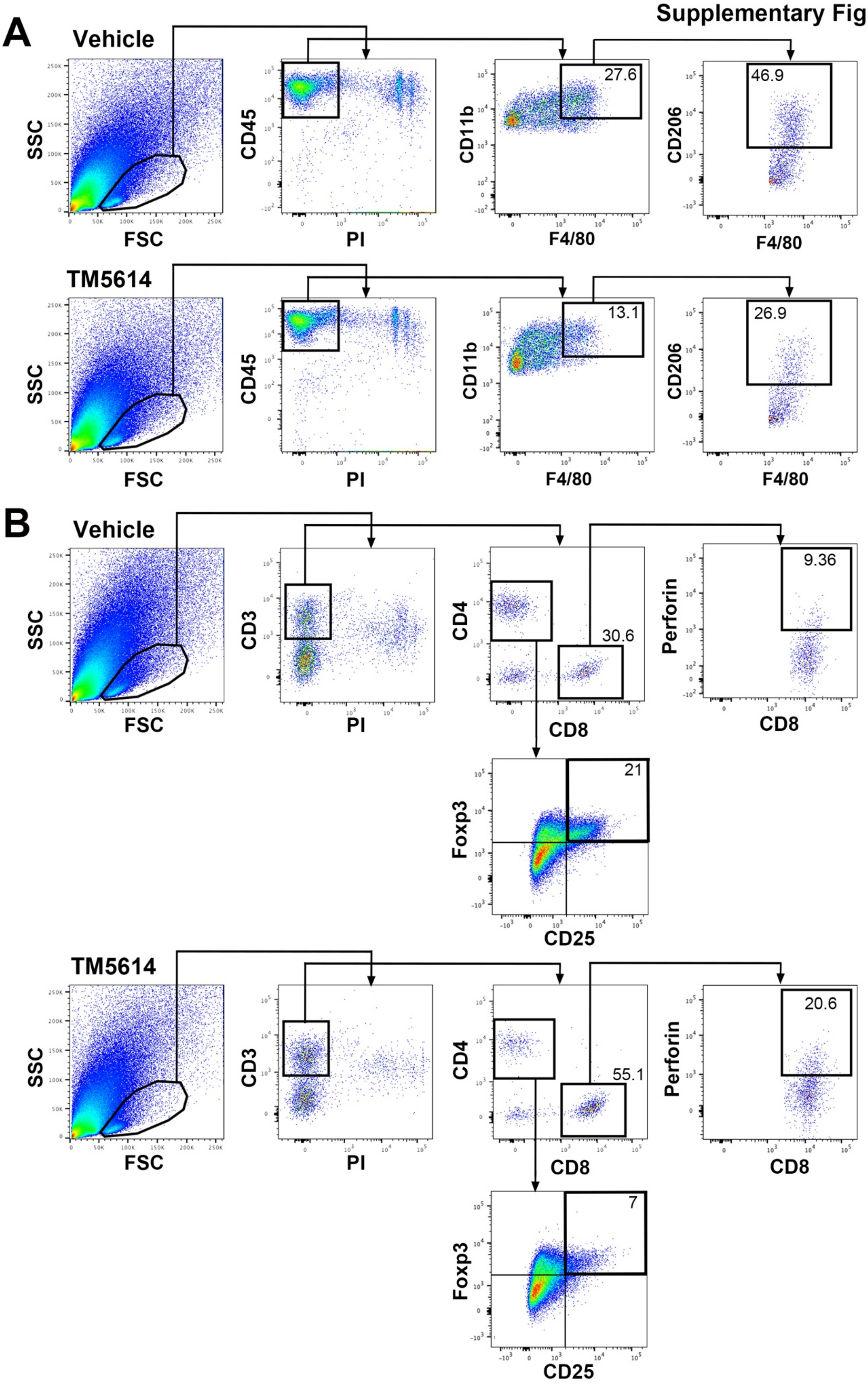

Supplementary Figure 4. Flow cytometry gating strategies; related to Figure 7. (**A**) Gating strategies for $CD11b^+F4/80^+CD206^+$ tumor-associated macrophages in tumor cells after administration of vehicle or TM5614 to the mice. (**B**) Gating strategies for $CD4^+$ T-cells, $CD8^+$ T-cells, and $CD4^+CD25^+Foxp3^+$ regulatory T-cells in the tumor cells after vehicle or TM5614 administration to the mice.

1.2 Supplementary Table S1

Primary Antibody	Clone / Cat. #	Manufacturer	Application
APC anti-mouse PD-L1 (CD274)	10F.9G2 / 124312	Biolegend	FC
Purified anti-mouse PD-L1 (CD274)	10F.9G2 / 124302	Biolegend	Blocking
AlexaFluor700 anti mouse CD3e	500A2 / 152316	Biolegend	FC
PE anti-mouse CD4	RM4-5 / 100512	Biolegend	FC
FITC anti-mouse CD8a	53-6.7 / 11-0081-85	eBioscience	FC
Alexa Fluor 700 anti-mouse CD69	H1.2F3 / 104539	eBioscience	FC
PE-Cy7 anti-mouse CD25	PC61 / 102016	Biolegend	FC
PE-Cy7 anti-mouse CD11b	M1/70 / 101216	Biolegend	FC
PE anti mouse CD206 (MMR)	C068C2 / 141706	Biolegend	FC
PE/Cy7 Annexin V	640950	Biolegend	FC
Rabbit anti-mouse PAI-1	ab28207	Abcam	FC
Rabbit anti-mouse PAI-1	ab66705	Abcam	FC
Recombinant human PAI-1	753804	Biolegend	CC
PE-Cy7 anti human/mouse Granzyme B	QA16A02 / 372213	Biolegend	FC
APC anti-mouse Perforin	S16009A / 154303	Biolegend	FC
PE-anti mouse IFN-gamma	XMG1.2 / 505808	Biolegend	FC
Rabbit anti-LRP1	EPR3724 / ab92544	Abcam	FC/blocking
Rabbit anti-uPAR	Ab103791	Abcam	FC
Rat anti-uPAR	109801 / MA5-23853	Invitrogen	FC/blocking
Rabbit anti Phospho-Jak1 (Y1034/1035) antibody	3331	Cell Signaling Technology	FC
Rabbit anti Phospho-Tyk2 (Tyr1054/1055) antibody	9321	Cell Signaling Technology	FC
Rabbit anti Phospho-Stat3 (Tyr705) antibody	9145	Cell Signaling Technology	FC
Mouse anti α-Smooth Muscle Actin	1A4 / A2547	Sigma-Aldrich	IHC
InVivoMab anti mouse PD-1	29F.1A12	Bio X Cell	In vivo

**Secondary Antibody**	**Clone / Cat. #**	**Manufacturer**	**Application**
PerCP/Cy5.5 Streptavidin	405214	Biolegend	FC
PE, Goat anti-Rat IgG	Poly4054 / 450406	Biolegend	FC
PE, Donkey anti-Rabbit IgG	Poly4064 / 406421	Biolegend	FC
Cy5 Streptavidin	405209	Biolegend	IF
PE, Alexa Flour647 anti-rabbit IgG	Poly4064 / 406421	Biolegend	FC
Alexa Fluor®488 goat anti rabbit IgG	A-11008	ThermoFisher Scientific	IF
Alexa Fluor®594 anti rabbit IgG	A-11012	ThermoFisher Scientific	IF

FC: Flow cytometry, CC: cell culture, IHC: Immunohistochemistry, IF: Immunofluorescence
